# Graft-versus-Host Disease after Living-Unrelated Kidney Transplantation

**DOI:** 10.1155/2014/971426

**Published:** 2014-04-09

**Authors:** N. Zacharias, M. H. Gallichio, D. J. Conti

**Affiliations:** Section of Transplantation, Department of Surgery, Albany Medical College, 47 New Scotland Avenue, Albany, NY 12208, USA

## Abstract

Graft-versus-host disease (GVHD) is a rare complication after solid organ transplantation and consists of a reaction of donor derived immune cells directed against host tissues. The vast majority of cases reported in the literature involve liver, small intestine and pancreas transplantation. We report a case of GVHD in a 48-year-old man after living-unrelated kidney transplantation at another center. Six months postoperatively he developed a skin rash, anorexia, and diarrhea that resulted in malnutrition and a 90 pound weight loss. At this point he was transferred to our center with a BMI of 16 and severe cachexia. Intravenous hyperalimentation was initiated and an extensive work-up for an infectious etiology was performed and was negative. An esophagogastroduodenoscopy was performed and revealed nodularity of the gastric mucosa, atrophy, and edema in the first and second portion of his duodenum. Biopsy findings were consistent with GVHD. Aggressive immunosuppressive therapy was instituted with a good response. The anorexia and diarrhea resolved, and he was discharged on hospital day 20. Three months later, there had been no recurrence of the diarrhea, the patient had gained an additional 40 pounds, BMI of 25, and a repeat upper endoscopy revealed complete resolution of the initial endoscopic abnormalities.

## 1. Introduction

Graft-versus-host disease (GVHD) is a rare, but often lethal, complication after solid organ transplantation (SOT) [1, 2]. It usually appears after transplantation of organs rich in immunocompetent cells such as the liver, small intestine and pancreas [2–4]. GVHD after SOT most commonly develops 1–11 weeks after transplant in one of two immunologic types [2]. The more common form is donor antibody mediated in blood groups A, B, or AB recipients who receive a solid organ from a blood group O donor [2]. The second type is cellular mediated which often affects the skin, liver, gastrointestinal tract, and bone marrow [2]. In this form, donor derived T-cells recognize and react to histoincompatible recipient cells of these organs [2–4]. The signs and symptoms are often subtle and include skin rash, fever, diarrhea, and hepatotoxicity [5]. In the posttransplant setting, these findings are usually attributed to opportunistic infections or immunosuppressive drug toxicity. Thus, the diagnosis of GVHD is often overlooked and delayed [5]. Histologic analysis of affected skin biopsies and bone marrow specimens is often used to make the diagnosis [5]. Fortunately, GVHD is extremely rare in kidney transplant patients. In our literature review we identified only 3 prior reports of GVHD after renal transplantation [1, 6, 7].

## 2. Case Report

**Figure 1:**
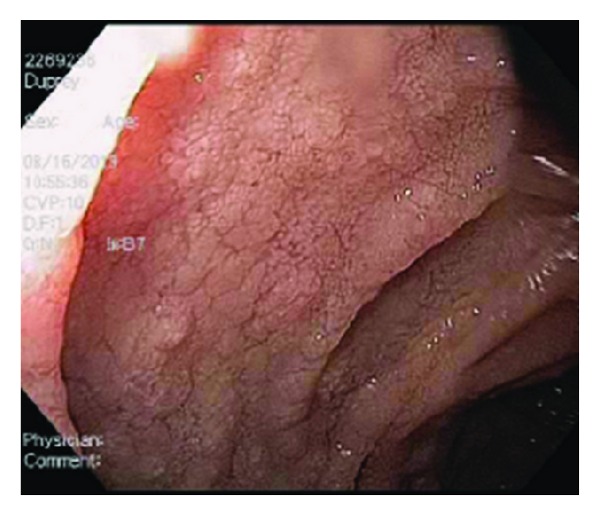
2nd portion of the duodenum: Nodularity, edema, atrophy.

A 48-year-old man with end-stage renal failure secondary to autosomal dominant polycystic kidney disease underwent a spousal living-unrelated renal transplant. His past medical history also included hyperlipidemia, cigarette smoking, gastroesophageal reflux, and an anxiety disorder. Six months after the transplant he developed a skin rash involving his neck and anterior chest and chronic unrelenting nonbloody diarrhea with associated anorexia, weight loss, malnutrition, and cachexia. Over the subsequent 18 months the patient had numerous hospitalizations with multiple changes in his immunosuppressive regimen without improvement and without a clear etiology of his syndrome being defined. During this period he lost 100 pounds and his body mass index dropped to 16. At this point he was transferred to our center for further evaluation and treatment. On admission his serum creatinine was 0.9 (mg/dL), WBC 1.8 (10³ U/L), hematocrit 23.7 (%), and platelet count 102,000 (10³ U/L). The serum liver function tests were normal. His immunosuppressive regimen on transfer included everolimus, tacrolimus, and prednisone. He was promptly started in total parenteral nutrition and the everolimus was discontinued. He underwent an extensive work-up including serum cytomegalovirus and Epstein-Barr virus PCR, serum cortisol level, clostridia difficile toxin PCR, cryptosporidium antigen, isospora stool culture, enteric pathogen stool culture, HIV testing, and hepatitis panel without any positive findings. A CT scan of his head, chest abdomen, and pelvis was positive only for bilateral ground-glass opacities of the lungs, unchanged from a chest CT scan 6 months priorly. Bronchoscopy with bronchoalveolar lavage culture and cytology was unremarkable. Bone marrow biopsy revealed a hypocellular matrix with no evidence of infection or malignancy. Colonoscopy revealed normal colonic mucosa and random biopsies were not helpful. However, at esophagogastroduodenoscopy he was found to have diffuse nodular edematous mucosa of the entire stomach, duodenal bulb, and second portion of the duodenum with patchy areas of atrophy involving both the gastric and duodenal mucosa (Figure 1). Biopsies of these areas were consistent with GVHD and included focal active duodenitis and increased epithelial apoptosis with focal crypt dropout. He was then treated with a 5-day course of Thymoglobulin, total dose 4 mk/kg, and intravenous methylprednisolone 250 mg/day for six consecutive days. The response to this treatment was dramatic. His diarrhea resolved within 4 days, his appetite improved, and he was able to tolerate an oral diet. He was subsequently discharged to home on tacrolimus, prednisone, and prophylactic valcyte tolerating an oral diet and gaining weight. Three months later he was feeling well with no recurrence of the diarrhea and had gained 65 pounds. In addition, a repeat upper endoscopy revealed complete resolution of the prior findings.

## 3. Discussion

The incidence of GVHD after renal transplantation is extremely rare but associated with significant morbidity and mortality [8]. Our patient was near death from malnutrition when we first became involved in his care. In reviewing the literature we identified only 3 prior cases of GVHD after renal transplantation alone [1, 6, 7] and seven after pancreas-kidney transplantation [4, 9–12].

Regarding the 3 cases of GVHD following renal transplantation alone, one resulted in death from multiorgan failure [7] and fortunately the other 2, as did our patient, responded to increased immunosuppressive therapy once the diagnosis was made. Similar to our case, all three previously reported patients presented with a skin rash and 2 developed gastrointestinal symptoms associated with duodenitis in their esophagogastroduodenoscopy [1, 6, 7]. Notably, in all cases, including ours, the diagnosis was delayed and was placed only after a thorough and complete work-up was performed that excluded other potential etiologies.

In the light of the consistently delayed diagnosis it is likely, but currently unclear, as to whether the outcome of GVHD can be improved by early diagnosis and treatment. A possible preventive measure for GVHD is graft irradiation, to reduce immunocompetent cell population, as it has been shown to decrease the incidence of GVHD after intestinal transplantation [13]. However, the incidence of GVHD is so rare after renal transplantation that the usefulness of this therapy is questionable and likely not cost effective.

In conclusion, we report a case of GVHD after living-unrelated (wife to husband) renal transplantation. To our knowledge this is the first case of living-unrelated kidney transplantation GVHD as the other three reported in the literature were a deceased donor transplant [7] and living-related transplants [1, 6]. In renal transplant patients who are present with these atypical symptoms in whom a more common diagnosis has not been arrived at after the usual medical evaluation, this entity, though rare, should be considered. Additional studies are necessary to develop effective methods for prevention, early diagnosis, and treatment.
